# The effects of tobacco taxation and pricing on the prevalence of smoking in Africa

**DOI:** 10.1186/s41256-021-00197-0

**Published:** 2021-04-29

**Authors:** Mustapha Immurana, Micheal Kofi Boachie, Abdul-Aziz Iddrisu

**Affiliations:** 1 Institute of Health Research, University of Health and Allied Sciences, Ho, Ghana; 2 Department of Health Policy Planning and Management, School of Public Health, University of Health and Allied Sciences, Ho, Ghana; 3 Banking Technology and Finance Department, Kumasi Technical University, Kumasi, Ghana

**Keywords:** Africa, Price, Smoking prevalence, Taxes, Tobacco, C33, H25, I12, I18

## Abstract

**Background:**

Tobacco use continues to kill millions of people globally, making it one of the major causes of preventable deaths. Notwithstanding, there has been a very marginal fall in the prevalence of tobacco smoking in Africa. Since taxes (hence prices) are part of the main measures suggested to decrease the demand for tobacco products, this study investigates how tobacco taxation and pricing influence the prevalence of smoking in 24 African countries.

**Methods:**

Using panel data on 24 African countries sourced from the World Health Organization (WHO) and the World Bank databases for the period 2010 to 2016, this study employs the system Generalized Method of Moments (GMM) estimator to investigate the effects of tobacco taxation and pricing on the prevalence of smoking. The system GMM estimator is used due its ability to deal with potential endogeneity of tobacco taxation and pricing: the likelihood that the prevalence of smoking can influence tobacco taxation and pricing which may lead to biased estimates.

**Results:**

Tobacco taxation and pricing have negative significant effects on the prevalence of smoking among the selected countries after controlling for growth of Gross Domestic Product (GDP) per capita, urbanization, death rate and net inflows of Foreign Direct Investment (FDI). Specifically, a percentage increase in tobacco price is found to decrease the prevalence of smoking by between 0.11 to 0.14%, while a percentage increase in tobacco tax decreases the prevalence of smoking by between 0.25 to 0.36%, all at 1% level of significance.

**Conclusion:**

Since tobacco taxation and pricing are found to have negative significant effects on the prevalence of smoking, the implication is that, their use can be intensified by African policy makers towards achieving the WHO Framework Convention on Tobacco Control (FCTC) recommended targets and hence decrease the prevalence of tobacco smoking in Africa. Doing so may therefore help in achieving the Sustainable Development Goal (SDG) 3.5 (prevention and treatment of substance abuse), thereby reducing the colossal number of smoking attributable deaths.

## Background

Morbidity and mortality resulting from tobacco use, although avoidable, remain high globally. It is estimated that, annually, lives lost to tobacco use surpasses eight (8) million worldwide [1]. Notwithstanding, about 1.3 billion people use tobacco, and a significant percentage of these people live in low- and middle-income countries [1, 2]. For the African region, it poses even a greater risk of tobacco use and related deaths in the future. This is evidenced in the rising number of people who use or smoke tobacco products. For example, 64 million (49 million), 65 million (51 million), 68 million (54 million) and 71 million (57 million) people were found to be using (smoking) tobacco in Africa in 2000, 2005, 2010 and 2015 respectively, while projections show that these figures would increase to 80 million (67 million) by the end of 2025.^1^ Thus, the African region remains part of the only three regions (including South-East Asia and Eastern Mediterranean regions) in which the number of tobacco smokers has been growing consistently. Moreover, the prevalence of current tobacco smoking in the African region has been declining only marginally (from 14.2% in 2000 to 10.8% in 2015). It is therefore, not surprising that, as of 2018, only eight African countries were likely to meet the World Health Organization (WHO) target of 30% relative reduction in the prevalence of tobacco use by 2025 (using 2010 as the reference year) [2]. The above situation has serious implications on productivity and healthcare systems, given that many non-communicable diseases are caused by tobacco use [3].

The staggering number of smokers and the associated deleterious effects have attracted attention from both academic and policy circles. Article 6 of the WHO Framework Convention on Tobacco Control (FCTC) recommends governments to use tax and price measures to control tobacco consumption [4]. By raising excise tax rates and/or imposing new ones, prices of tobacco products (all else being equal) would rise [5]. This makes smoking cost-prohibitive; deters potential smokers; and encourages cessation as well as engender a cutback in the quantum consumed by current smokers [5–8]. 

Whether the strategy of using tax and price measures to curb smoking yields the desired results, is an empirical question that has received considerable attention in the literature [7–16]. Among these studies, only Ho et al. [11] conducted a cross-country analysis for Africa, and found cigarette prices to decrease cigarette consumption. However, a common limitation in many of these studies is the use of techniques that fail to address the potential endogeneity of tax or price: the likelihood that smoking or using tobacco products may also influence tobacco taxes or prices. As a result, many of the estimates in these studies may be essentially biased.

Although some studies [11, 16] addressed the potential endogeneity of prices, the authors (like most of the other empirical studies) considered the effect of price on *quantity* of tobacco products (*cigarette in the case of these studies*) consumed or smoked and not the *prevalence* of tobacco use or smoking. While the quantity consumed informs us about the amount of tobacco products consumed in a period, it does not reveal the percentage of the population involved (i.e., participation). However, the prevalence measure of tobacco use reveals the percentage of the population who use tobacco products. This measure gives a broader understanding of the proportion of the population that is at risk of developing smoking-related diseases and premature deaths [17]. Therefore, reducing prevalence has more public health benefits. In addition, the prevalence measure is more useful for time series or cross-country analysis since data on prevalence of smoking is more readily available relative to the quantity smoked [18]. Further, many of the studies conducted their analyses for only cigarettes. However, tobacco products such as shisha and cigar are also used in Africa.

This study, therefore, investigates the effects of tobacco taxation and pricing on the prevalence of smoking in 24 selected African countries. The study makes a significant contribution to this strand of the literature generally and the African context in particular. We deal with the endogeneity problem by using the system Generalized Method of Moments (GMM) estimator, and consider the prevalence of tobacco smoking as opposed to cigarette smoking (*focusing on only quantity*). Doing so reveals results that are robust and unbiased, which can be employed in the African context for effective policymaking as regards using taxes and prices to control tobacco use.

## Methods

### Data sources

This study uses data from 2010 to 2016^2^ on 24 African countries (see Table 7 in Appendix). Data on tobacco tax and price are obtained from the WHO [19], while data on all the remaining variables are from the World Bank [20]. The number of countries and the study duration are mainly determined by data availability, especially regarding tobacco tax and price as well as the prevalence of smoking.

### Variables

The dependent variable is the prevalence of smoking (Smoking) and the main independent variables are tobacco tax (Tax) and price (Price). The control variables used are growth rate of Gross Domestic Product (GDP) per capita (GDP per cap.), urban population growth rate (Urbanization), death rate (Death rate) and Foreign Direct Investment (FDI).

Tobacco tax refers to the total tax on a pack of 20 cigarettes. This tax includes import duties, excise taxes, Value Added Tax (VAT) and other applicable taxes expressed as a percentage of the retail price of the most sold brand. Price of tobacco refers to the retail price of a pack of 20 of the most sold brand of cigarette in international dollars (at Purchasing Power Parity (PPP)) [19]. We use cigarette taxes and prices to proxy taxes and prices for all tobacco products because of data unavailability pertaining to taxes and prices for all other tobacco products. Moreover, cigarettes are the most used tobacco products [21].

Death rate is measured per 1000 people. Smoking prevalence is measured as the percentage of population (≥ 15 years) who currently smoke any tobacco product (excluding the use of smokeless tobacco), whether on daily or non-daily basis in a year. Growth of GDP per capita is measured by the yearly growth rate of GDP divided by (midyear) population, measured in percentages. Urbanization refers to the growth rate of the number of people living in urban areas on an annual basis, measured in percentages. FDI is measured by investment (the net inflows) to obtain a permanent managerial interest (voting stock of ≥10%) in a country that differs from the investor’s country, expressed as a percentage of GDP [20].

Regarding the expected signs of the explanatory variables, based on the theory of demand, it is expected that both tobacco price and tax will have negative effects on the prevalence of smoking. This is because rising taxes would increase the prices of tobacco products and hence decrease the amount of tobacco that people can smoke as well as the number of people who take up smoking or raise the number of quitters, ceteris paribus [6]. The sign of GDP per capita may be negative or positive depending on whether tobacco is a normal good or an inferior good. If tobacco is a normal good, an increase in income is expected to increase the prevalence of smoking, while prevalence of smoking will decline if tobacco is an inferior good. Urbanization is expected to have a positive effect on the prevalence of smoking [see 22–24], because it can serve as a good market for tobacco firms. Also, rural dwellers who travel to urban areas as part of urbanization may give up some cultural norms (which view smoking to be a deviant behavior) associated with rural areas to adopt smoking, which is seen as a typical urban lifestyle. We expect death rate to have a negative effect on the prevalence of smoking because rising mortalities (especially preventable deaths) may force people to adopt healthy behaviors such as quitting smoking. FDI is expected to have a positive effect on the prevalence of smoking because more FDI inflows may be associated with increased production and consumption of tobacco [25].

### Empirical model

Equation 1 is used to estimate the effects of tobacco tax and price on the prevalence of smoking:

1 $$ {\mathrm{Smoking}}_{\mathrm{it}}=\mathrm{f}\ \left({\mathrm{T}}_{\mathrm{it}},{\mathrm{X}}_{\mathrm{it}},{\upmu}_{\mathrm{it}}\right) $$

where T is a vector of tobacco tax and price, X indicates a set of control variables, *i* represents country, *t* represents time and μ indicates the error term. Smoking is as already defined.

Equation 1 is re-specified more formally by including the first lag of the dependent variable (to take care of the persistence as well as the initial level of prevalence of smoking) and time fixed effects as follows:

2 $$ {\mathrm{Smoking}}_{\mathrm{it}}={\upalpha}_0+{\upalpha}_1{\mathrm{Smoking}}_{\mathrm{it}-1}+{\upalpha}_2{\mathrm{T}}_{\mathrm{it}}+{\upalpha}_3{\mathrm{X}}_{\mathrm{it}}+{\Omega}_{\mathrm{t}}+{\upmu}_{\mathrm{it}} $$

where α_0_ is the intercept of the regression equation, the remaining α_s_ are coefficients of their respective variables, Smoking_it−1_ is the first lag of the prevalence of smoking, and Ω_t_ represents time fixed effects (year dummies).

### Data analysis

In estimating Eq. 2, one major challenge is the possibility of endogeneity. First, there is the possibility of smoking prevalence (dependent variable) influencing right hand side variables (independent or explanatory variables) such as tobacco tax, tobacco price and death rate. For instance, high smoking prevalence may push governments to increase excise taxes and/or impose new ones which in turn raises prices of tobacco, in order to decrease tobacco consumption (participation and intensity). Moreover, rising prevalence of smoking may increase future deaths rates. Second, the inclusion of the first lag of the prevalence of smoking in Eq. 2 may lead to endogeneity because it may correlate with the error term [26–28]. If these endogeneity concerns are not addressed, they may lead to biased and unreliable estimates, since the coefficients of the independent or explanatory variables may not be true reflections of their effects on the dependent variable. Given the above, this study uses the system GMM estimator by Arellano and Bover [29] and Blundell and Bond [30] as the empirical data analysis technique. The system GMM estimator addresses endogeneity by using first differenced and level regressions as well as lags of the explanatory variables as instruments. The system GMM therefore provides a means of using the second-order serial correlation and Hansen overidentification tests to verify the validity of the instruments used. Moreover, the system GMM is more suitable for datasets with many cross-sections but few time periods [27], as is the case in this study.

## Results

### Descriptive statistics

In this sub-section, we present the average smoking prevalence, tobacco taxes and prices for the selected countries (see Table 1). Changes in smoking prevalence, tobacco taxes and prices between 2010 and 2016 (see Table 2) as well as summary of the variables (see Table 3) are also presented.

**Table 1:** Mean smoking prevalence, tobacco taxation and pricing per country (2010–2016)

Country	Smoking (%)	Tax (%)	Price (Int. US$ at PPP)
Algeria	15.357	48.703	3.211
Benin	6.8	8.933	2.289
Botswana	19.743	52.494	6.739
Burkina Faso	13.014	32.759	2.506
Cape Verde	9.643	23.84	3.794
Rep. of the Congo	20.229	38.2	2.448
Ethiopia	4.429	27.6	1.686
Gambia, The	16.186	47.586	2.098
Ghana	4.157	25.411	2.871
Kenya	11.614	52.746	2.684
Mali	12.357	26.661	3.434
Mauritius	22.171	72.059	6.745
Mozambique	17.571	28.123	1.584
Namibia	21.443	44.884	6.427
Niger	7.4	30.714	2.023
Nigeria	6.029	20.63	2.555
Rwanda	13.086	31.514	2.007
Senegal	8.557	33.286	1.98
Seychelles	22.214	71.465	11.276
South Africa	20.657	49.567	5.725
Togo	7.714	11.506	1.888
Uganda	11.057	44.831	2.174
Zambia	14.471	35.796	2.919
Zimbabwe	16.114	38.529	2.241

Smoking prevalence and tax are in percentages; price is in international dollars (at PPP)

**Table 2:** Trend in smoking prevalence, tobacco taxation and pricing per country (2010–2016)

Country	Smoking (%)		Tax (%)		Price (Int. US$ at PPP)	
	2010	2016	2010	2016	2010	2016
Algeria	15.2	15.6	48.78	43.06	3.22	4.62
Benin	7.2	6.4	11.96	6.42	2.37	2.31
Botswana	19.7	20	55.41	49.72	4.95	8.83
Burkina Faso	13.6	12.5	32.2	34.81	2.45	3.06
Cape Verde	10.2	9.1	23.84	23.84	3.73	3.87
Rep. of the Congo	14.7	26.9	35.5	40.94	1.87	3.78
Ethiopia	4.5	4.4	39.23	18.77	1.67	1.8
Gambia, The	16.9	15.5	56.06	54.09	1.02	3.21
Ghana	4.4	3.9	22.23	28.1	3.19	3.61
Kenya	12.5	10.7	63.79	52.25	3.16	2.82
Mali	12.5	12.3	21.85	27.69	3.66	3.67
Mauritius	22.6	21.6	71.71	70.23	4.79	7.86
Mozambique	18.7	16.6	26.53	31.39	1.26	1.8
Namibia	21.5	21.4	46.22	43.13	5.88	7.3
Niger	7.1	7.7	29.62	35.27	1.85	2.27
Nigeria	6.3	5.8	20.63	20.63	2.89	2.26
Rwanda	13.9	12.3	29.44	54.73	2.02	1.36
Senegal	9	8.2	30.25	37.75	1.73	2.27
Seychelles	22.9	21.5	67.57	70.76	11.08	14.49
South Africa	21	20.3	52.94	52.4	4.81	5.68
Togo	8.1	7.4	10.73	10.58	1.86	2.19
Uganda	12.2	10	44.59	50.85	2	2.44
Zambia	15.2	13.8	36.29	37.32	2.09	4.81
Zimbabwe	16.4	15.8	51.69	35.9	1.01	3.49
Africa (average for all the 24 countries)	13.60	13.32	38.71	38.78	3.11	4.16

Smoking prevalence and tax are in percentages; price is in international dollars (at PPP)

**Table 3:** Summary statistics of the prevalence of smoking, tobacco price, tobacco tax and control variables for all countries (2010–2016)

Variable	Obs	Mean	Std. Dev.	Min	Max
Smoking	168	13.417	5.776	3.9	26.9
Price	168	3.471	2.352	1.01	14.49
Tax	168	37.41	16.132	6.42	79.71
GDP per cap.	168	2.667	3.306	−10.862	18.066
Urbanization	168	3.572	1.408	−1.912	6.177
Death rate	168	8.578	2.114	4.656	14.27
FDI	168	5.29	8.059	−1.032	57.838

Smoking prevalence and tax are in percentages; price is in international dollars (at PPP); GDP per cap., Urbanization and FDI are in percentages, while death rate is per 1000 people

From Table 1, it is evident among the selected countries that Seychelles (22.214%), Mauritius (22.171%), Namibia (21.443%), South Africa (20.657%), Republic of the Congo (20.229%) and Botswana (19.743%) are the six countries with the highest smoking prevalence rates over the sampled period. With regard to tobacco taxation, Mauritius (72.059%), Seychelles (71.465%), Kenya (52.746%), Botswana (52.494%), South Africa (49.567%) and Algeria (48.703%) are the six countries with the highest tobacco taxes. Also, Seychelles (11.276), Mauritius (6.745), Botswana (6.739), Namibia (6.427), South Africa (5.725) and Cape Verde (3.794) are the six countries with the highest tobacco prices (in international dollars at PPP).

Thus, Mauritius, Seychelles, Botswana and South Africa being among the top countries with high tobacco taxation and pricing is understandable given that they are part of the countries with the highest prevalence of smoking. The implication is that these taxes and prices might have been raised in order to reduce the relatively high prevalence of smoking in these countries.

Also, Ghana (4.157%) and Ethiopia (4.429%) are the two countries with the lowest smoking prevalence. Further information on the prevalence of smoking, taxation and pricing can be seen in Table 1.

From Table 2, it can be seen that while the prevalence of smoking in countries such as Algeria, Botswana and Republic of the Congo increased between 2010 and 2016, most of the countries experienced decreasing prevalence of smoking, though very marginal. Also, all the countries in the sample (except Benin, Kenya, Nigeria and Rwanda) experienced increases in tobacco prices. Therefore, it is worth investigating whether tobacco prices have played any role in the marginal reduction in the prevalence of smoking experienced in most of the countries.

In Table 3, the summary statistics of the variables used in the study are presented. It can be seen that the prevalence of smoking, tobacco price, tobacco tax, growth rate of GDP per capita, urban population growth, death rate and FDI net inflows as a percentage of GDP have mean values of 13.42%, Int. US$3.47, 37.41%, 2.67, 3.57%, 8.58 per 1000 people and 5.29% respectively. Thus from 2010 to 2016, 13.42% of the population (≥ 15 years) in the selected countries smoked tobacco. This calls for the use of mechanisms such as taxation and pricing to reduce the number of people using tobacco products. Notwithstanding, the average overall tax of 37.41% is far less than the WHO recommended level of more than 75% [21], hence making it worrying regarding the fight against the use of tobacco products in Africa.

### Correlation analyses of prevalence of smoking, tobacco price, tobacco tax and control variables

In this sub-section, the correlation analyses of variables (see Table 4) are presented.

**Table 4:** Matrix of correlations of the prevalence of smoking, tobacco price, tobacco tax and control variables (2010–2016)

Variables	(1)	(2)	(3)	(4)	(5)	(6)	(7)
(1) Smoking	1.000						
(2) Price	0.621	1.000					
(3) Tax	0.724	0.648	1.000				
(4) GDP per cap.	−0.072	− 0.042	0.034	1.000			
(5) Urbanization	−0.470	−0.470	− 0.435	−0.128	1.000		
(6) Death rate	−0.050	−0.141	−0.298	0.117	0.250	1.000	
(7) FDI	0.222	0.070	−0.004	0.030	−0.034	0.081	1.000

In Table 4, the correlation matrices between both tobacco tax and price, and smoking prevalence are not less than 0.6 which show the strength of relationships between these variables and hence the need for further analysis using a multivariate approach. Moreover, the correlation matrices among the independent variables are low^3^ which indicate the less likelihood of  multicollinearity.

### Results of the effects of tobacco tax, tobacco price and control variables on smoking prevalence

This section presents results of the system GMM regressions on the effects of tobacco tax and price on smoking prevalence in the 24 selected African countries (Tables 5 and 6). It should be noted that in all estimations, the standard errors are robust to heteroscedasticity and autocorrelation. Moreover, the second-order serial correlation tests (AR (2)) and the Hansen overidentification tests show the absence of second-order serial correlation and overidentification respectively, hence, confirming the appropriateness of the instruments used. Also, the overall *p*-values of all our regression results are statistically significant at 1%. These tend to affirm the efficiency and unbiasedness of our results as well as their implications. 

In Table 5 where the effect of tobacco prices on the prevalence of smoking is examined, as expected, the lag of the dependent variable is statistically significant. Regarding price, we find that it has a negative significant effect in all the models. Specifically, price is found to have coefficients of − 0.43, − 0.44 and − 0.52 in Models 1, 2 and 3 respectively. These coefficients are all significant at 1%. Thus, a unit increase in the price of tobacco products is found to decrease the prevalence of smoking by 0.43, 0.44 and 0.52 percentage points in Models 1, 2 and 3 respectively. Using elasticities,^4^ a percentage increase in price decreases the prevalence of smoking by 0.11% in Models 1 and 2 and by 0.14% in Model 3. Further, urbanization is found to have a positive significant effect on the prevalence of smoking in Model 3. Specifically, a unit increase in urban population growth is found to increase smoking prevalence by 0.39 percentage points in Model 3 at 10% level of significance.

**Table 5:** The effect of tobacco price on the prevalence of smoking

	Model 1	Model 2	Model 3
L.Smoking	1.30^***^	1.31^***^	1.38^***^
	(0.07)	(0.07)	(0.10)
GDP per cap.	0.07	0.07	0.09
	(0.05)	(0.05)	(0.08)
Urbanization	0.28	0.31	0.39^*^
	(0.23)	(0.23)	(0.22)
Price	−0.43^***^	− 0.44^***^	− 0.52^***^
	(0.10)	(0.11)	(0.14)
Death rate		−0.06	− 0.02
		(0.24)	(0.16)
FDI			−0.04
			(0.03)
Constant	−3.78^**^	−3.65^*^	−4.99^**^
	(1.54)	(1.93)	(2.11)
Observations	144	144	144
Countries	24	24	24
Instruments	15	16	17
AR(2)	−0.90	−0.97	−0.85
AR(2) *p*-value	0.37	0.33	0.40
Hansen	4.88	4.82	2.43
Hansen *p*-value	0.43	0.44	0.79
Wald chi2	680.90	951.53	1253.67
Wald chi2 *p*-value	0.00	0.00	0.00

L.Smoking refers to the first lag of smoking prevalence; Death rate and FDI are introduced in Models 2 and 3 to show the robustness of the effect of price; AR (2) refers to second-order serial correlation test; Hansen refers to the test for overidentification; Robust standard errors in parentheses; ^*^ *p* < 0.1, ^**^ *p* < 0.05, ^***^ *p* < 0.01; For brevity, year dummies are not reported

In Table 6, we present the results of the effect of tobacco taxation on the prevalence of smoking tobacco. As anticipated, we find the lag of the dependent variable to be significant in all models. Also, the coefficient of tax is negatively significant in all the models. Specifically, tobacco taxation is found to have respective coefficients of − 0.09 (Models 1 and 2) and − 0.13 (Model 3) that are significant at 1%. The implication is that, a unit increase in tobacco tax decreases the prevalence of smoking by 0.09 percentage points in Models 1 and 2, as well as by 0.13 percentage points in Model 3. Interpreting the tax coefficients as elasticities, we find that a percentage increase in tax decreases the prevalence of smoking by 0.25% in Models 1 and 2 and by 0.36% in Model 3.

**Table 6:** The effect of tobacco tax on the prevalence of smoking

	Model 1	Model 2	Model 3
L.Smoking	1.38^***^	1.36^***^	1.51^***^
	(0.09)	(0.06)	(0.16)
GDP per cap.	0.08^*^	0.07^***^	0.11^**^
	(0.04)	(0.03)	(0.04)
Urbanization	0.23	0.25	0.40
	(0.19)	(0.17)	(0.25)
Tax	−0.09^***^	−0.09^***^	−0.13^***^
	(0.03)	(0.02)	(0.05)
Death rate		−0.29	−0.42^**^
		(0.24)	(0.19)
FDI			−0.06
			(0.04)
Constant	−2.90^**^	−0.28	0.20
	(1.39)	(2.32)	(2.15)
Observations	144	144	144
Countries	24	24	24
Instruments	15	16	17
AR(2)	−0.63	−0.66	0.74
AR(2) p-value	0.53	0.51	0.46
Hansen	5.59	6.16	3.22
Hansen p-value	0.35	0.29	0.67
Wald chi2	554.88	2118.66	3212.68
Wald chi2 p-value	0.00	0.00	0.00

L.Smoking refers to the first lag of smoking prevalence; Death rate and FDI are introduced in Models 2 and 3 to show the robustness of the effect of tax; AR (2) refers to second order-serial correlation test; Hansen refers to the test for overidentification; Robust standard errors in parentheses; ^*^ *p* < 0.1, ^**^ *p* < 0.05, ^***^ *p* < 0.01; For brevity, year dummies are not reported

As regards the control variables, GDP per capita is found to have positive coefficients of 0.08, 0.07 and 0.11. These coefficients are statistically significant at 10, 1 and 5% in Models 1, 2 and 3 respectively. Thus, a unit increase in GDP per capita is found to increase smoking prevalence by 0.08, 0.07 and 0.11 percentage points in Models 1, 2 and 3 respectively. Moreover, death rate is found to have a coefficient of − 0.42 in Model 3, which is significant at 5%. Thus, a unit increase in death rate decreases smoking prevalence by 0.42 percentage points.

## Discussion

### Effects of tobacco tax and price on the prevalence of smoking

The findings of the current study are in line with the existing literature. As regards the price of tobacco, its finding of decreasing the prevalence of smoking in all the models is not surprising since higher prices can make tobacco products cost-prohibitive which will deter or reduce smoking [5-8]. Previous studies have found results similar to this [7, 11, 16]. In South Africa, hikes in cigarette prices resulting from excise tax increases led to a significant decline in cigarette consumption [31]. Consistent with the literature [6], the demand for tobacco products is price inelastic since all the coefficients are less than one. This can be attributed to the addictive nature of tobacco use such that, even in the presence of a price increment, some people would still be willing to smoke. The implication is that, price increments should be very high in order to make a significant reduction in the prevalence of smoking. Moreover, other measures such as banning tobacco advertising and smoking in public as well as creating awareness of the harmful effects of tobacco, could complement tobacco tax in the attempt to combat smoking. This is because it has been found that, exposure to legacy, state-sponsored as well as pharmaceutical advertisement was associated with less smoking in the United States [32], whiles a ban on tobacco advertising or promotion was found to reduce awareness of tobacco promotions in the United Kingdom [33]. Also, findings from 22 Organization for Economic Co-operation and Development [OECD] countries showed that using a set of comprehensive bans on tobacco advertising can decrease the consumption of tobacco [34]. Further, in Bangladesh, a ban on indoor smoking at worksites was found to enhance the likelihood of quitting smoking [35].

Similar to price, the finding that tobacco taxation reduces the prevalence of smoking is not farfetched because, an increase in tax would make tobacco products expensive through its effect on price [5]. This finding buttresses the conclusion that taxes are potent in decreasing the consumption of tobacco among low-income groups of which Africa is not an exception [6, 21]. Similarly, Levy et al. [10] and Sharbaugh et al. [12] found a rise in tax to decrease the prevalence of smoking in Taiwan and the United States respectively.

### Effect of GDP per capita on the prevalence of smoking

With regard to income, we find that an increase in GDP per capita increases the prevalence of smoking (Table 6). This is because as people’s incomes increase, they are more capable of affording other products (such as tobacco) beyond the basic necessities of life. The results show that tobacco is a normal good and that growing income makes it more affordable. This finding is in tandem with previous studies that found rising incomes to influence tobacco consumption positively [9, 15]. Given the continent’s (Africa) impressive growth performance for some time now, the current smoking prevalence in Africa is unlikely to fall substantially, if efforts are not bolstered. For instance, between 2002 and 2008, the average economic growth rate for Africa was roughly 5.6% [36]. In 2013, Africa’s economic growth rate of 3.7% exceeded the global economic growth rate of 2.4%. The same can be said for the year 2014 [37]. It is therefore not surprising that Blecher and Ross [18] posit that increasing economic growth has increased the number of smokers as well as the quantity of cigarettes smoked in Africa.

### Effect of urbanization on the prevalence of smoking

The findings show that, the prevalence of smoking increases when urban population grows. This outcome is not surprising since rising urban population may be seen by the tobacco industry as a potential market for tobacco trade, hence, would be attracted to these urban centers. Moreover, people from rural areas who travel to urban areas as part of urbanization may adopt lifestyles such as smoking that are more typical of urban areas relative to rural areas, which may increase the prevalence of smoking. This finding can be related to previous studies that found rising rural population to decrease the consumption of cigarette among a sample of African countries [11], as well as moderate and high urbanization (urbanicity) increasing smoking attitude among black women in South Africa [22]. Also, it has been found that urban females have five times higher smoking prevalence than their rural counterparts in Thailand [23], while students from urban settings were more likely to initiate smoking relative to those from rural settings in Thailand [24]. These findings provide signals with regard to the need to pay critical attention to urban areas concerning policies aimed at reducing the consumption of tobacco.

### Effects of FDI and death rate on the prevalence of smoking

FDI is found to have a negative but insignificant effect on the prevalence of smoking. This conflicts the finding of Gilmore and McKee [25] who found FDI to increase the production and consumption of tobacco. Our finding can be attributed to the fact that in recent times, FDI inflows into Africa are more concentrated in the services sector relative to the manufacturing sector [38]. Moreover, the negative sign of FDI (though insignificant) can be related to Immurana [39] who found FDI to improve health in Africa.

Also, as expected, death rate is found to decrease the prevalence of smoking. This is not farfetched since rising death rate may force people to adopt healthy lifestyles such as avoiding smoking. In fact, information on smoking-attributed deaths may induce behavioral change among the population.

### Limitations

The study is not without limitations. In examining the effects of taxes and prices on tobacco smoking prevalence, the ideal situation would have been to use taxes and prices for all tobacco products instead of cigarette prices and taxes. Moreover, our study is restricted to a sample of 24 African countries and hence may be limited in terms of generalizing the findings to represent the African continent, given that there are more than 50 countries on the continent. Also, tax and price measures are just some of the many tobacco control measures. For instance, restrictions on smoking in public places and sale to minors; public health campaigns on smoking, comprehensive bans on promotion and advertising; and health warning labels may also help to control tobacco use. It would have therefore been useful if the study examined such policies to find the extent to which they decrease the prevalence of smoking.

## Conclusion

This study examines the effects of tobacco taxation and pricing on the prevalence of smoking in 24 African countries for the period, 2010–2016, while controlling for per capita income, urbanization, death rate and FDI. The system GMM estimator is used as the empirical data analysis technique. We find that while rising per capita income and urbanization have positive significant effects on the prevalence of smoking, the effect of death rate is negatively significant. Notwithstanding, it must be noted that these control variables were only significant in some of the models. With regard to tobacco taxes and prices, we find that they have negative significant effects (at 1% level) on the prevalence of smoking, even after robustness checks. The implication is that, tobacco taxation and pricing help in decreasing the prevalence of smoking in Africa. This buttresses the point by the WHO that tobacco tax and price measures are effective tools in reducing the prevalence of smoking. The findings call for African governments to adhere to the WHO FCTC strategies, especially those on taxes, in order to significantly reduce the prevalence of tobacco use. Doing so would help in curbing the huge number of deaths attributed to tobacco use and hence help in achieving the Sustainable Development Goal (SDG) 3.5 (prevention and treatment of substance abuse) and to a greater extent SDG 3 (good health and wellbeing).

## Data Availability

The data used for the study are freely available from the websites of the World Health Organization (https://www.who.int/tobacco/global_report/2017/appendix-ix/en/) and the World Bank (https://databank.worldbank.org/source/world-development-indicators).

## Appendix

**Table 7:** List of countries

Algeria	Mozambique
Benin	Namibia
Botswana	Niger
Burkina Faso	Nigeria
Cape Verde	Rwanda
Rep. of the Congo	Senegal
Ethiopia	Seychelles
Gambia, The	South Africa
Ghana	Togo
Kenya	Uganda
Mali	Zambia
Mauritius	Zimbabwe

## Abbreviations

FCTC: Framework Convention on Tobacco Control
FDI: Foreign Direct Investment
GDP: Gross Domestic Product
OECD: Organization for Economic Co-operation and Development
PPP: Purchasing Power Parity
SDG: Sustainable Development Goal
WHO: World Health Organization
GMM: Generalized Method of Moments
VAT: Value Added Tax

## Footnotes

1 The figures not in brackets represent those who use tobacco and those in the brackets represent those who smoke tobacco.

2 We fill gaps in the data with linear interpolation.

3 Apart from tobacco tax and price because they are not put together in the same model.

4 As suggested by a reviewer, the elasticities are obtained by multiplying the price (tax) coefficients by the ratio of the mean price (tax) to the mean smoking prevalence.
